# Nursing interventions on self-care and quality of life among Indian women with abnormal uterine bleeding

**DOI:** 10.6026/973206300210025

**Published:** 2025-01-31

**Authors:** Sabari Raja, Theranirajan Ethiraj, Shankar Shanmugam Rajendran, Valveeman Vijayalakshmi, Asvini Murugesan, Seethalakshmi Durairaj

**Affiliations:** 1 Department of Obstetrics & Gynaecology, College of Nursing, Madras Medical College, The TN Dr MGR Medical University, Chennai, Tamil Nadu, India; 2 Department of Pediatrics, Madras Medical College, The TN Dr MGR Medical University, Chennai, Tamil Nadu, India; 3 Department of Pediatric Nursing, Madras Medical College, The TN Dr MGR Medical University, Chennai, Tamil Nadu, India

**Keywords:** Abnormal uterine bleeding, nursing intervention, self-care, quality of life, women's health

## Abstract

Abnormal Uterine Bleeding (AUB) affects 10-30% of women of reproductive age, often leading to physical and emotional distress. Therefore, it is of interest to evaluate the effectiveness of selected nursing interventions in improving self-care and quality of life among Indian women with AUB at a maternity tertiary care centre in Chennai using a quasi-experimental non-randomized control group design. Nursing interventions, including education on symptom management, lifestyle modifications and personalised support, significantly improved self-care and quality of life in the experimental group. A comparison of pre-test and post-test scores confirmed the effectiveness of these interventions with demographic factors influencing patient outcomes. Thus, the importance of structured nursing interventions in enhancing symptom management and overall well-being in women with abnormal uterine bleeding is reported.

## Background:

Women play a crucial role in society, encompassing a wide range of responsibilities that go beyond their physical attractiveness. Women frequently play a vital role in society, serving as the foundation of communities, promoting beneficial transformations and cultivating an environment of inclusiveness [1]. They have a critical impact on education, healthcare and social services, influencing the development of future generations. Women persistently strive to dismantle structural obstacles as champions of equal rights, guaranteeing a fairer and more just world for everyone [2]. The uterus represents women's fortitude and tenacity. This factor is crucial in women's general health and well-being, influencing their lives, including their menstrual cycle and menopause experience. The uterus possesses the remarkable capacity to generate, support and rejuvenate life, rendering it an organ of tremendous importance and marvel within the human body [3]. Abnormal uterine bleeding, characterized by irregular or excessive bleeding, impacts around 10-30% of women throughout their reproductive years. Tackling these issues typically necessitates medical intervention, which can encompass a spectrum of treatments, including pharmaceuticals and surgical procedures, contingent upon the gravity and characteristics of the ailment [4]. Abnormal uterine bleeding (AUB) is a prevalent gynecological condition that impacts 10-30% of women in their reproductive years. It is distinguished by erratic, excessive, or extended menstrual bleeding. Abnormal uterine bleeding (AUB) can occur due to a range of factors, such as hormone imbalances, fibroids, polyps, or abnormalities affecting the lining of the uterus. The unpredictability and inconvenience of bleeding disrupt daily activities and work, leading to stress and decreased quality of life [5, 6]. Self-care is crucial for women with abnormal uterine bleeding to manage their symptoms and maintain their well-being. Studies found that 65% of women who practiced self-monitoring of their bleeding patterns and adhered to prescribed medication regimens reported a significant improvement in their symptoms [7, 8]. Nurses play a crucial role in educating women with abnormal uterine bleeding (AUB) by providing comprehensive information about the condition, its symptoms and management strategies. They offer guidance on self-care practices, such as monitoring bleeding patterns, managing pain and adopting healthy lifestyle habits. Therefore, it is of interest to evaluate the effectiveness of selected nursing interventions in improving self-care and quality of life among Indian women with abnormal uterine bleeding at a maternity tertiary care centre in Chennai using a quasi-experimental, non-randomized control group design.

## Materials and Methods:

This study employed a Quantitative research approach, a Quasi-experimental, non-randomised control group design, to investigate the effects of selected nursing interventions on self-care and quality of life in women with abnormal uterine bleeding at a maternity Tertiary care centre in Chennai. The study lasted four weeks.

## Quantitative phase:

Women diagnosed with Abnormal uterine bleeding aged between 35 and 55 who understand and read Tamil and are willing to participate in the study were chosen using a probability purposive sampling technique. The exclusion criteria include women with any other medical or psychiatric illness and those already participating in another intervention study. Self-care Assessment tool covers multiple areas: physical, psychological, emotional, spiritual, relational and professional self-care, providing a simple method for identifying areas where they are thriving and those needing attention. The WHO QOL-BREF scale, a 26-item questionnaire, measures four domains of quality of life: physical health, psychological health, social relationships and environment.

## Ethical considerations:

The Institutional Ethics Committee of Madras Medical College, Chennai-03, ensures that all research procedures adhere to recognised moral standards. Additionally, written permission was obtained from the Institute of Obstetrics and Gynaecology directors in Egmore, Chennai-08. These approvals underscored the commitment to uphold ethical research practices, including protecting participant privacy, informed consent and the right to withdraw from the study at any time.

## Results:

Recent studies have highlighted the potential benefits of nursing interventions in improving women's self-care and quality of life with abnormal uterine bleeding For instance, a survey by Shaban *et al.* (2024) found that an educational intervention delivered by nurses significantly improved self-care behaviours and quality of life among women with abnormal uterine bleeding Another study by Agarwal *et al.* (2023) reported that a nurse-led self-management program resulted in a 50% reduction in symptom severity and a 40% improvement in quality-of-life scores among participants. Educational interventions play a crucial role in improving self-care and quality of life for women with abnormal uterine bleeding (AUB). A study by Kanagasabai *et al.* (2023) evaluated an educational program that provided women with abnormal uterine bleeding information on symptom management, lifestyle modifications and treatment options. The results showed that 80% of participants reported increased knowledge and confidence in managing their condition, improving their quality of life scores [9].

## Demographic variables of the study participants:

Age distributions were similar, with the majority between 41-45 years at 40% in the experimental group and 36% in the control group. Most participants were married (94% and 92%, respectively) and belonged to nuclear families (66% and 70%). Educational levels were predominantly primary (40% and 38%). The majority were unskilled workers (54% in both groups). Income was mostly below Rs.9307 for 70% of the experimental and 62% of the control groups. Most lived in urban areas (66% and 60%, respectively) and the diet was primarily non-vegetarian (86%). Religion was predominantly Hindu (58% and 62%, respectively) and family members were the primary source of social support (92% and 84%, respectively). Chi-square tests indicated no statistically significant differences between the groups across these variables.

## Clinical variables of the study participants:

Clinical variables of study participants show that most participants started menstruating between ages 13 and 15, comprising 74% of the experimental and 70% of the control group. Menstrual cycles mostly fell within 21-35 days, accounting for 72% and 76%, respectively. Most participants experienced menstrual periods lasting 3-5 days (62% experimental, 64% control). The typical menstrual flow involved using five sanitary pads daily for 38% of the experimental and 36% of the control groups. The most common number of living children was one (60% experimental, 64% control) and the majority had no miscarriages (72% experimental, 76% control). Chi-square tests showed no statistically significant differences across these variables.

Table 1 compared the experimental group's domain-wise pre-test and post-test mean quality of life (QOL) scores. Significant improvements were observed across all domains following the intervention. The Physical and Psychological domains showed substantial increases of 18.90 and 18.15 points, respectively. The Social Domain experienced a notable improvement of 21.04 points. While starting with the lowest baseline, the Environmental Domain saw an increase of 24.91 points. The total mean quality of life score of the group rose from 43.96 to 64.66, marking a significant overall improvement of 20.70 points. All changes were statistically significant, with p-values ≤ 0.001, indicating robust improvements in the experimental group's quality of life As shown in Figure 1 (see PDF), the experimental group demonstrated significantly higher posttest scores across all domains compared to the control group, highlighting the effectiveness of the intervention.

Table 2 analysed the changes in domain-wise mean quality of life (QOL) scores within the control group from the pre-test to the post-test. Minor improvements were noted in all domains. The Physical and Psychological domains each saw a marginal increase of 0.84 points. The Social Domain experienced a slightly higher improvement of 2.91 points and the Environmental Domain increased by 1.99 points. The total mean quality of life score for the group modestly rose from 43.73 to 45.37, an overall gain of 1.64 points. However, none of these changes was statistically significant, as indicated by p-values ranging from 0.08 to 0.17, suggesting that the control group's quality of life scores remained relatively stable without substantial interventions.

## Discussion:

First objective was to assess the pretest level of self-care and quality of life among Women with Abnormal uterine bleeding in experimental and control groups. The present study revealed initial disparities in self-care and quality of life between the experimental and control groups during the pretest phase. In the experimental group, 48% displayed low levels of self-care, compared to 44% in the control group. Moderate self-care levels were similar, at 38% and 40%, respectively, with high self-care slightly lower at 14% versus 16%. Regarding quality of life the experimental group had 66% of participants with poor quality of life somewhat less than the control group's 68%. Both groups showed comparable moderate quality of life levels, at 34% for the experimental and 32% for the control, with no participants in the good quality of life category. The findings of the present study were supported by another study conducted by Youssif *et al.* (2022), who reported that 45% of women in their experimental group and 47% in the control group had low self-care levels before any intervention, closely mirroring the results of 48% and 44%, respectively [10].

Second Objective was to evaluate the effectiveness of selected nursing intervention on self-care and quality of life among Women with Abnormal uterine bleeding in the experimental group. The current study demonstrated the impact of a nursing intervention on self-care and quality of life among participants. The experimental group's self-care scores improved significantly, rising from 53.61% to 74.47%, a gain of 20.86%, while the control group saw a marginal increase from 54.37% to 55.13%, a gain of only 0.76%. Similarly, quality of life scores in the experimental group surged from 43.73% to 64.66%, reflecting a 20.70% improvement. In contrast, the control group experienced a slight increase in quality of life from 43.96% to 45.37%, a gain of 1.64%. These outcomes highlight the effectiveness of the selected nursing intervention in enhancing both self-care and quality of life The present study's findings were supported by another study conducted by Kanagasabai *et al.* (2023), who reported a study which saw an increase in self-care scores by 18% and quality of life by 19.5% following a nursing intervention [9].

Third Objective as to compare the pretest and post-test levels of self-care and quality of life among Women with Abnormal uterine bleeding in the experimental and control groups. The present study demonstrated marked improvements in self-care and quality of life after a targeted intervention. Self-care gains were significant across domains, particularly in the Balance domain, which saw a 7.60-point increase, leading to an overall mean score rise of 29.20 points. Regarding quality of life the most significant improvements were observed in the Environmental domain, with a 24.91-point increase, followed by the Social domain, which improved by 21.04 points. The overall mean quality of life score advanced from 43.96 to 64.66, a rise of 20.70 points. All changes were statistically robust, with p-values ≤ 0.001, indicating the intervention's effectiveness in substantially enhancing the experimental group's health outcomes. H states that there was a statistically significant difference between the pre and post-intervention level of self-care and quality of life among women with Abnormal Uterine Bleeding in both the experimental group and the control group. Hence, H1 was accepted. Fourth objective was to find out the association of posttest level of self-care and quality of life among Women with Abnormal uterine bleeding with their selected demographic variables in the experimental group The present study identified a significant association between demographic characteristics and enhancements in self-care and quality of life following a targeted intervention. Women aged 41-45 showed the highest self-care proficiency, with 85% achieving high scores, especially those from joint families, where 82.35% excelled. Women with menstrual cycles of 21-35 days also had notable high self-care scores (72.22%). Regarding quality of life the same age group had a higher likelihood of attaining good quality of life scores (60%) and a similar positive trend was observed among those earning between Rs.9308 and Rs.27,882, with 61.54% showing improved quality of life These findings highlight the influence of age and socioeconomic factors on health outcomes post-intervention. The present study's findings were compared with another study conducted by Vitale *et al.* (2022), which reported that abnormal uterine bleeding women aged 40-45 had the highest improvement in quality of life similar to this finding of 60% in the same age group [11]. H2 states a statistically significant association in the post-intervention level of self-care and quality of life among women with Abnormal Uterine Bleeding in the experimental group with their selected demographic variables. Hence, H2 was accepted. Almuhaitb *et al.* highlighted that nursing interventions significantly enhance self-care practices and improve the quality of life in women with abnormal uterine bleeding. Personalized education and supportive care play a crucial role in symptom management and overall well-being [12].

## Conclusion:

Nursing interventions focused on education and personalised care is crucial for improving self-care and quality of life among women with AUB. This study emphasises the importance of specialised training and skills development in gynaecological nursing, ensuring that nurses are equipped to address the complex needs of patients with gynaecological conditions. However, the study also acknowledges the need for further research to refine these interventions and explore their long-term benefits across diverse populations. Moving forward, nursing practices and educational programs must evolve continually, integrating the latest research and innovative practices to uphold the high standards of care required in gynaecological nursing.

## Figures and Tables

**Table 1:** Comparison of domains pre-test and post-test mean quality of life score among experimental group

**Domains score**	**Group**				**Mean**	**Student**
	**Pre-test**		**Post-test**		**difference**	**paired t-test**
	**Mean**	**SD**	**Mean**	**SD**		
Physical Domain	48.23	12.9	67.13	10.7	18.9	t=11.25 p=0.001***(S)
Psychological Domain	45.18	14.9	63.33	16	18.15	t=12.53 p=0.001***(S)
Social Domain	43.13	18.6	64.17	25.6	21.04	t=7.30 p=0.001***(S)
Environment Domain	39.28	17.2	64	24.9	6.36	t=7.90 p=0.001***(S)
Pretest Total	43.96	13.4	64.66	18.1	7.6	t=11.90 p=0.001***(S)
p≤0.001 very high significant S=significant

**Table 2:** Comparison of domain wise pre-test and post-test mean quality of life score among control group

**Domains score**	**Group**				**Mean**	**Student**
	**Pre-test**		**Post-test**		**difference**	**paired t-test**
	**Mean**	**SD**	**Mean**	**SD**		
Physical Domain	48.63	13.7	49.47	15.2	0.84	t=1.75 p=0.08(NS)
Psychological Domain	45.58	15.5	46.42	17	0.84	t=1.76 p=0.08(NS)
Social Domain	42.33	20.1	45.24	15.6	2.91	t=1.37 p=0.17(NS)
Environment Domain	38.37	22.6	40.36	22.4	1.99	t=1.55p=0.16(NS)
Pretest Total	43.73	15.7	45.37	14	1.64	t=1.44 p=0.15(NS)
P>0.05 not significant
NS=not significant

## References

[1] Lwamba E (2022). Campbell Syst Rev..

[2] Hurdle D.E (2001). Health & Social Work..

[3] Peacock K (2025). Menopause..

[4] Deligeoroglou E, Creatsas G (2012). Endocr Dev..

[5] Wouk N, Helton M (2019). Am Fam Physician..

[6] Jewson M (2020). Best Pract Res Clin Obstet Gynaecol..

[7] Marnach M.L, Laughlin-Tommaso S.K (2019). Mayo Clin Proc..

[8] Selmi C, Marca A.L (2023). Eur J Contracept Reprod Health Care..

[9] Kanagasabai P.S (2023). Int J Gynaecol Obstet..

[10] Youssif A.M (2022). Egyptian Journal of Health Care..

[11] Vitale S.G (2022). Diagnostics..

[12] Almuhaitb R.A (2024). J Clin Med..

